# Case Report: Delivery or not, generalized arterial calcification of infancy in China

**DOI:** 10.3389/fmed.2026.1761482

**Published:** 2026-04-14

**Authors:** Yanxiu Guo, Jing Wang, Ying Gu, Qiang Ma, Li Lin

**Affiliations:** 1 Department of Gynaecology and Obstetrics, Peking University International Hospital, Beijing, China; 2 Department of Gynaecology and Obstetrics, Ultrasonic diagnosis group, Peking University International Hospital, Beijing, China; 3 Department of Pathology, Peking University International Hospital, Beijing, China

**Keywords:** China, GACI, generalized arterial calcification of infancy, orphan diseases, prenatal diagnosis

## Abstract

Generalized arterial calcification of infancy (GACI) is an extremely rare, often fatal condition characterized by widespread calcification and stenosis of both large and medium-sized arteries. The majority of cases are caused by biallelic variants in the *ENPP1* gene, leading to deficiency of an enzyme critical for preventing pathological calcium deposition in arterial walls. The prognosis of GACI is severe, with fetal and infant mortality rates reaching up to 55%. This study reports a GACI case diagnosed at 24 weeks in a 32-year-old woman with a history of miscarriages, whose fetal ultrasound at 24 weeks revealed striking echogenic foci and thickening in the heart and major blood vessels. This prompted a multidisciplinary team to suspect GACI. Following confirmation through amniocentesis and given the poor prognosis, the parents opted to terminate the pregnancy at 26 weeks. This case underscores the critical role of advanced prenatal imaging and genetic analysis in diagnosing GACI. Ultimately, management decisions extend beyond medical science, involving profound ethical and personal considerations for families facing this devastating diagnosis.

## Introduction

Generalized arterial calcification of infancy (GACI) is a rare condition characterized by extensive arterial calcification and stenosis of both large and medium-sized arteries. The estimated incidence of GACI is only 1 in 200,000 people (1), and only 200 cases have been reported in the literature since its first description in 1899. GACI has a high mortality rate of approximately 55% (1–3) during the fetal period and infancy; however, there are documented cases of survival into adulthood. The majority of GACI cases (approximately 75%) are caused by biallelic variants in the gene that encodes ectonucleotide pyrophosphatase/phosphodiesterase-1 (*ENPP1*), leading to *ENPP1* enzyme deficiency (2–4). Less commonly, GACI (approximately 9–10%) is caused by variants in the ATP-binding cassette sub-family C member 6 (*ABCC6*) (3, 5, 6). Mutations in the *ENPP1* gene can lead to decreased inorganic pyrophosphate (PPi) levels and hydroxyapatite crystal production that are deposited in various layers of arteries, including the aorta, coronary arteries, heart valves, and renal arteries, causing vascular lesions, soft tissue calcification around joints, and various life-threatening conditions due to vascular lesions such as myocardial infarction, renal failure, cerebral infarction, and hypertensive encephalopathy. Approximately half of GACl cases (48%) are diagnosed in utero, and most of the other half (52%) are diagnosed at a median age of 3 months (7). GACI diagnosis may entail a combination of clinical manifestations, imaging, histopathologic findings, and genetic testing.

## Case report

The report was approved by the ethics committee of Peking University International Hospital, and informed consent was obtained by the recruited couple.

In March, 2025, a 32-year-old woman, gravida 3 para 0, was admitted to the medical center at 26 weeks + 1 day of gestation due to her abnormal ultrasonic inspection report. Her first two pregnancies ended in miscarriage due to fetal arrest at 8 weeks and 9 weeks, respectively. The fetus did not receive any laboratory inspection after the first miscarriage in April, 2022. Before the second pregnancy, the patient and her husband underwent chromosomal testing. Her karyotype was identified as 46, XX, 1qh+. A microscopic analysis of 30 metaphase cells confirmed a female karyotype with an increase in heterochromatin on the long arm of one chromosome 1. Her husband’s karyotype was 46, XYqh-. The microscopic analysis of 30 metaphase cells revealed a male karyotype showing reduced heterochromatin on the long arm of the Y chromosome. After the second miscarriage in November 2023, genomic analysis of the abortion specimen was performed using low-pass whole-genome sequencing (LP-WGS) via next-generation sequencing (NGS), and the analysis revealed that the chromosomes of the fetus were normal.

The patient was born and raised in northern China, with no history of smoking, alcohol consumption, or exposure to toxic chemicals, radiation, or infectious diseases during pregnancy. Her family had no three-generation history of hereditary diseases, cardiovascular calcification diseases, recurrent miscarriages, or perinatal fetal death. The patient’s parents and siblings were in good health with no abnormal cardiovascular imaging findings. The patient’s husband also had no significant personal or family medical history, and the couple did not have a consanguineous marriage.

To investigate the etiology of her recurrent miscarriages, she underwent a series of laboratory tests after the second miscarriage. She was diagnosed with Hashimoto’s thyroiditis in 2023 and has been on an oral Euthyrox therapy since then. She was also diagnosed with atypical antiphospholipid syndrome (APS) due to elevated levels of β2-glycoprotein I antibodies (aβ2GPI, 26.07 RU/mL), anti-protein S antibodies (aPS, 29.2 RU/mL), and anti-phosphatidylserine/prothrombin IgM antibodies (aPS/PT IgM, 42.72 RU/mL), before her third pregnancy.

The patient reported regular menstrual cycles occurring every 30 days, with a flow duration of 7 days. Her last menstrual period (LMP) was documented on 8 September, 2024, with a positive urine pregnancy test 30 days post-LMP. A first-trimester ultrasound confirmed a gestational age consistent with the LMP, establishing an estimated delivery date of 15 June, 2025. During early gestation, she experienced significant nausea, which was managed with oral vitamin B6. Due to documented low progesterone levels, she received combined progesterone therapy—oral capsules and intramuscular injections—until 10 weeks of gestation. For atypical APS, anticoagulation was initiated with aspirin at a dose of 50 mg daily until 13 weeks of gestation, followed by a dose escalation to 100 mg daily through 25 weeks. Concurrent immunomodulatory therapy included hydroxychloroquine, which was started at 6 weeks and discontinued after 10 weeks. Supplemental anticoagulation with subcutaneous low-molecular-weight heparin (bemiparin sodium) at a dose of 3,500 IU daily was administered from approximately 8 weeks until 10 weeks of gestation.

The ultrasonography performed at 12 weeks showed that nuchal translucency was 0.12 cm, which is considered normal. At 13 and 14 weeks of gestation, the patient underwent screening for high-risk genetic disorders, including cell-free DNA (cfDNA) testing through maternal peripheral blood sampling, both of which yielded normal results. Fetal movements were first perceived at 20 weeks of gestation. An ultrasound at 22^+4^ weeks indicated a gestational age of 21^+5^ weeks, revealing suboptimal maternal weight gain; therefore, nutritional supplementation was advised. During the 24th gestational week, a 75 g oral glucose tolerance test (OGTT) returned values of 5.2 mmol/L (fasting), 5.4 mmol/L (1-h), and 6.9 mmol/L (2-h), establishing a diagnosis of gestational diabetes mellitus (GDM). Through dietary modifications and exercise, glycemic control was maintained within the target ranges: fasting blood glucose levels of 4.7–4.9 mmol/L and postprandial levels of 5.7–6.5 mmol/L.

**Figure 1:**
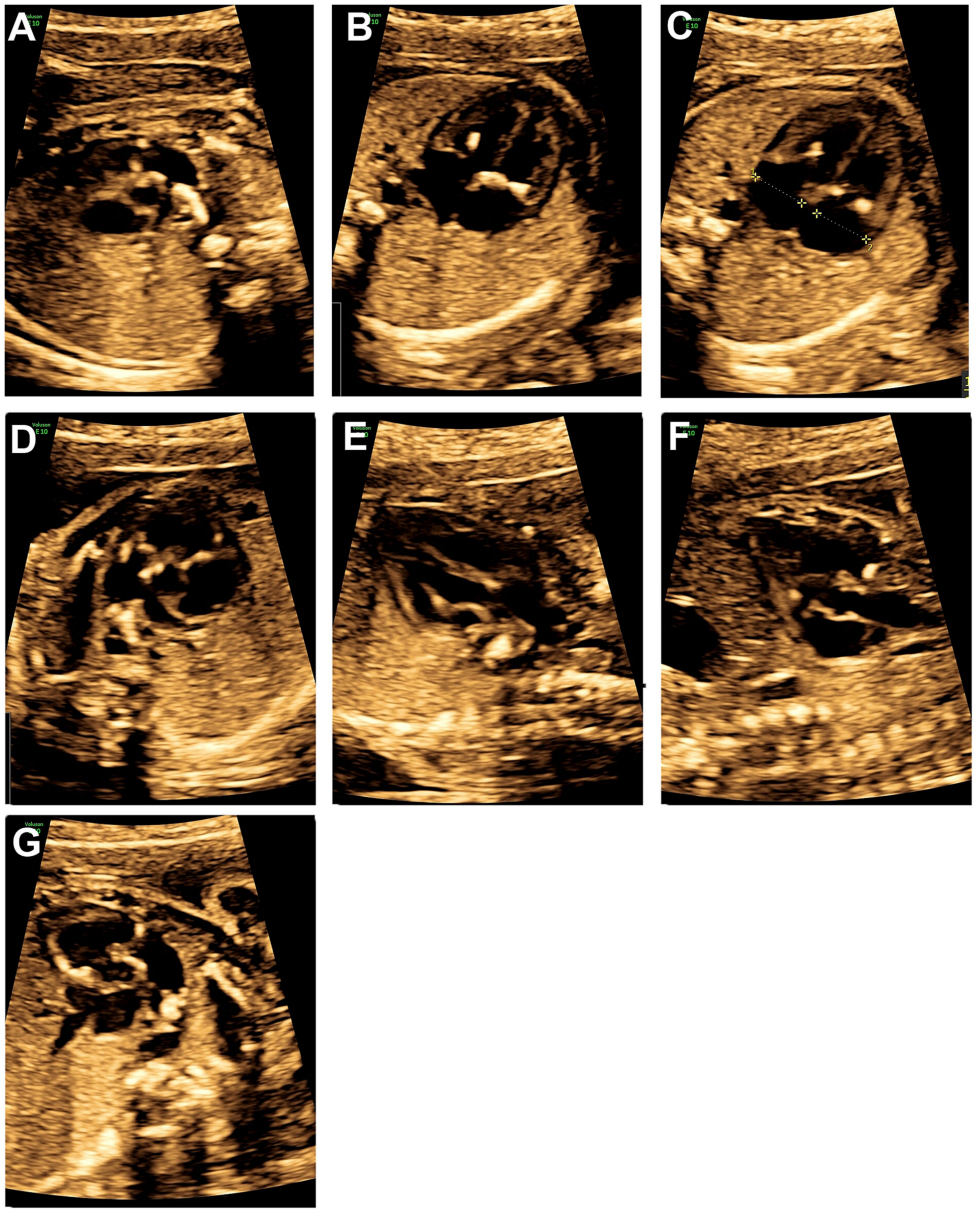
Fetal echocardiogram at 24 weeks. **(A)** Calcification of pulmonary valve, main pulmonary artery and ductus arteriosus wall, **(B)** calcification of the tricuspid valve and left ventricular chordae tendineae, **(C)** calcification of the mitral valve apex, **(D)** calcification of the septum of the main pulmonary artery trunk, **(E)** calcification of the aortic origin, **(F)** calcification of the aortic valve and vessel wall, **(G)** calcification of the walls of the heart valves and great vessels.

**Figure 2:**
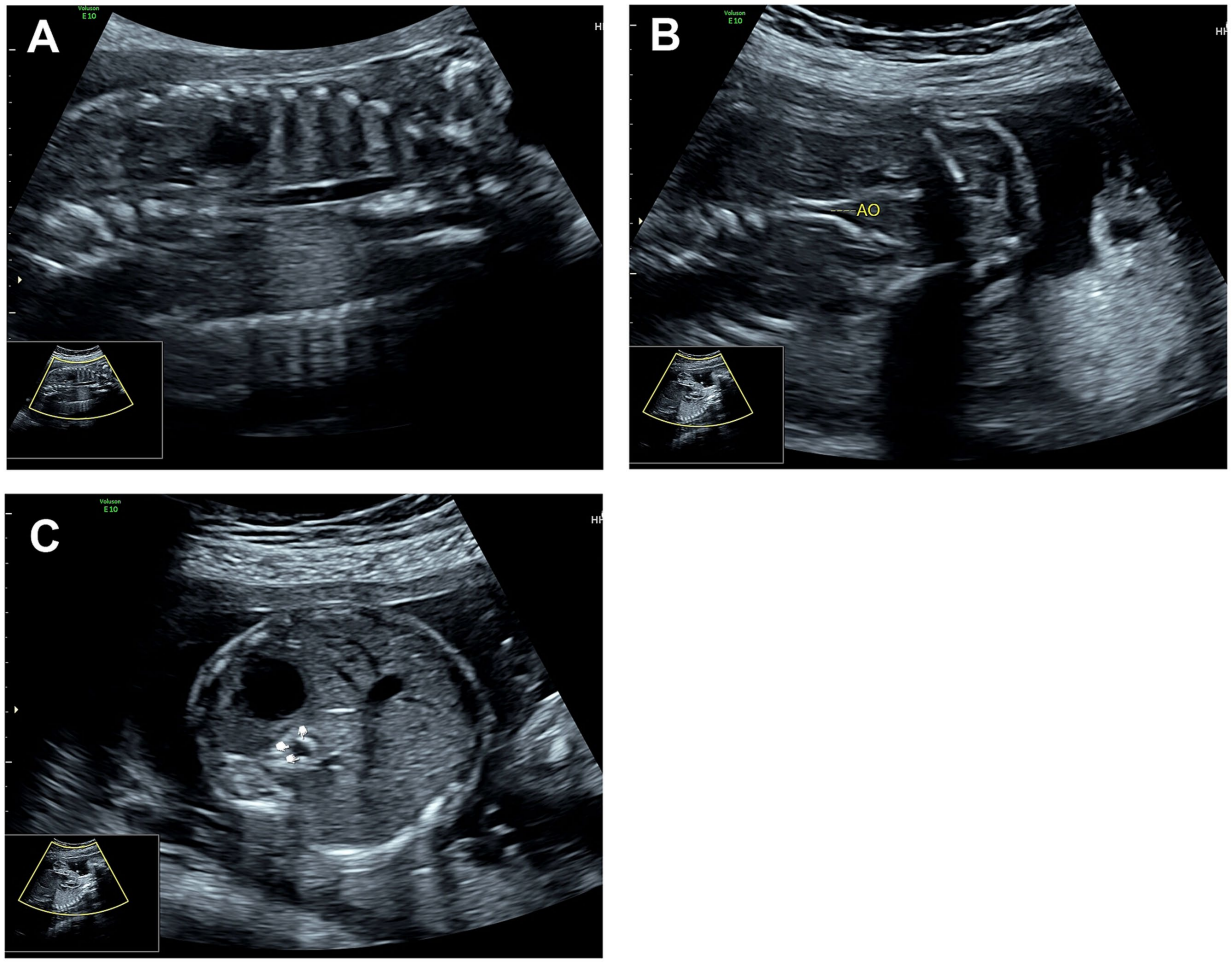
Fetal echocardiogram. **(A)** Calcification of the descending aortic wall, **(B)** Calcification of the descending aorta and iliac arteries, **(C)** The cross-sectional view of the descending aorta shows thickening and calcification of the vessel wall.

The ultrasonic cardiogram (UCG) of the fetus at 24 weeks reported local thickening and enhanced echogenicity in the heart and the walls of major blood vessels: pulmonary valve, main pulmonary artery and ductus arteriosus wall (Figure 1A), tricuspid valve and left ventricular chordae tendineae (Figure 1B), mitral valve (Figure 1C), the septum of the main pulmonary artery trunk (Figure 1D), the aortic origin (Figure 1E), the aortic valve and vessel wall (Figure 1F), and the walls of the heart valves and great vessels (Figure 1G). The transverse diameter of the right ventricular cavity was slightly smaller than the internal diameter of the tricuspid valve annulus, and the internal diameter of the left ventricular tricuspid valve annulus was normal; the inner diameter of the aortic isthmus was slightly narrowed. There was a mild regurgitation in both the mitral and tricuspid valves. Diffuse thickening, enhanced echogenicity, and luminal narrowing were also observed in the walls of bilateral iliac arteries, the abdominal aortic septum, and the distal segment of the abdominal aorta (Figures 2A–C).

**Figure 3:**
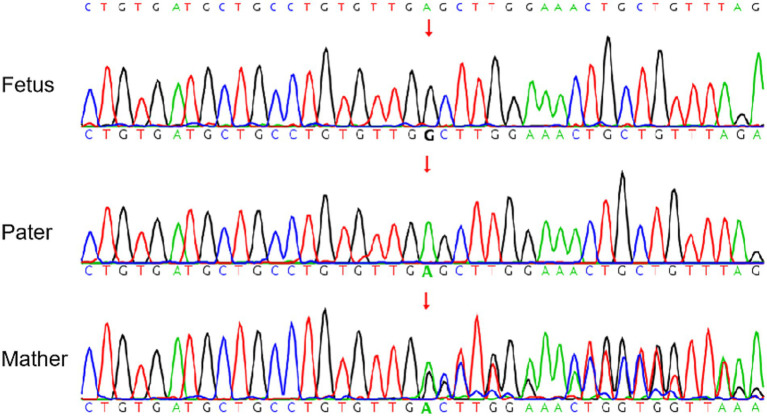
SNP test of the fetus and parents. *ENPP1*: NM_006208.3:c.383del (p.E128Gfs*7) mutation was detected in the fetus while the pater has a wild type phenotype and the mother has a heterozygous phenotype.

According to the abnormal UCG feature and the abnormal development of the cardiovascular system of the fetus, the clinicians highly suspected that the fetus had GACI, after the discussion with the multidisciplinary team (MDT). To test this hypothesis, amniocentesis and whole exome sequencing on both the cells from the amniotic fluid and the blood cells from both parents were performed. The results of the whole exome sequencing showed that the fetus had a homozygous *ENPP1* gene mutation on chromosome 6, which might have been inherited from his mother due to maternal uniparental disomy (Figure 3).

Based on the UCG test and *ENPP1* mutation, the fetus was ultimately diagnosed with GACI. After careful consideration, the patient and her family have opted to terminate the pregnancy at 26 + 2 weeks due to the poor fetal prognosis.

**Figure 4:**
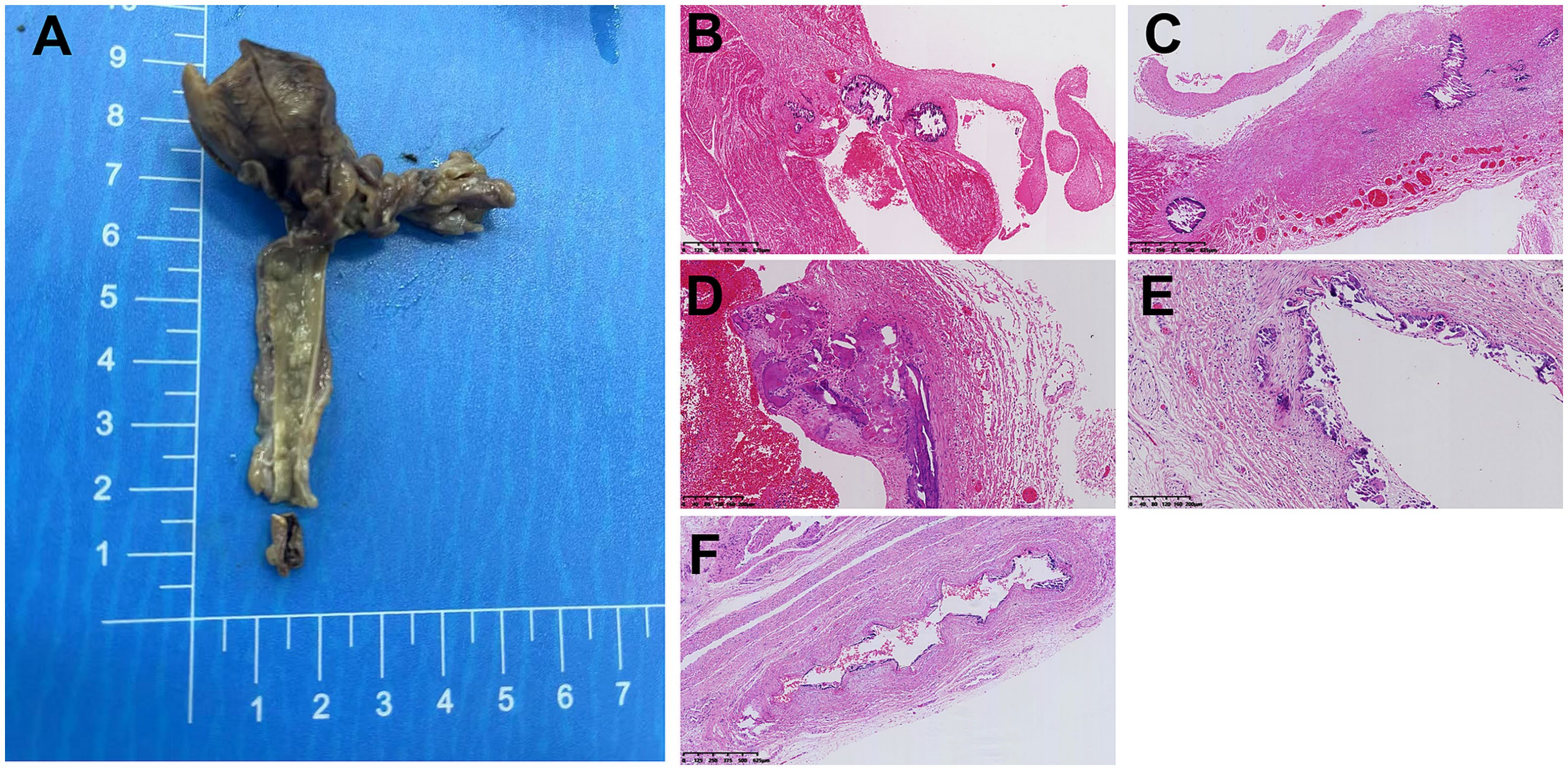
**(A)** The gross specimen of the aorta shows a grayish-yellow plaque, with a size of 4 × 0.7 cm. **(B)** Calcification foci can be observed beneath the endocardium of the left ventricle and beneath the endocardium of the right ventricle. **(C)** Patchy calcification can be seen beneath the aortic intima and within the vessel walls. **(D,F)** Calcification can also be observed beneath the intima of the carotid artery, laryngeal artery and intravesical artery.

An autopsy was conducted after the operation. The HE staining results of the tissue sections revealed significant calcification foci beneath the endocardium of both the left and right ventricles, beneath the aortic intima and within the vascular wall, beneath the intima of the laryngeal arteries, and beneath the intima of arteries within the bladder wall (Figure 4). The final fetal autopsy results confirmed the diagnosis of GACI.

## Discussion

GACI, also historically known as Idiopathic Arterial Calcification of Infancy (IACI), is an extremely rare and fatal systemic vascular calcification disorder. GACI is definitely characterized as an autosomal recessive genetic disease, meaning an affected child must inherit one pathogenic allele from each parent to develop the condition. Additionally, uniparental disomy of the relevant chromosome is a primary cause of the disease when only one parent carries the gene. GACI exhibits significant genetic heterogeneity, primarily associated with mutations in two genes.

*ENPP1* gene (GACI type 1). This is the most predominant causative gene, accounting for approximately 67–75% of diagnosed cases due to biallelic loss-of-function mutations (2–4). *ENPP1* is responsible for generating extracellular inorganic pyrophosphate (PPi), a crucial physiological inhibitor of mineralization (2, 3, 5).

*ABCC6* gene (GACI type 2). About 9–10% of cases are caused by mutations in the *ABCC6* gene (3, 5, 6). The protein encoded by *ABCC6* is thought to be involved in the transport of extracellular adenosine triphosphate (ATP), which serves as the substrate for *ENPP1* to produce PPi (3–5). Notably, *ABCC6* mutations are also the primary cause of another calcification disorder, Pseudoxanthoma Elasticum (PXE).

Other genes and unknown mutations Approximately 24% of cases show no mutations in *ENPP1* or *ABCC6*, suggesting the existence of other unknown pathogenic genes or mechanisms (3, 8). For example, mutations in the *NT5E* gene can cause Calcification of Joints and Arteries (CALJA), a less severe adult-onset disease related to GACI (2, 3).

GACI is a multisystem disease whose core manifestations stem from progressive calcification and stenosis of systemic arteries. Arterial calcification and stenosis are the hallmark feature of GACI. Calcification begins in the internal elastic lamina of the large and medium-sized arteries, extending into the intima and media, often accompanied by intimal fibroproliferation, leading to severe luminal narrowing (4, 5, 9). The most commonly affected arteries include the aorta, coronary arteries, pulmonary arteries, renal arteries, and mesenteric arteries (2, 3, 5). Widespread arterial calcification causes a drastic reduction in vascular compliance, triggering refractory hypertension, which is the root cause of other complications (10–12). Congestive heart failure is one of the most common causes of death. Coronary artery calcification can directly lead to myocardial ischemia and infarction. Concurrently, systemic hypertension and pulmonary hypertension (due to pulmonary artery involvement) significantly increase cardiac afterload, ultimately resulting in severe, rapidly progressive congestive heart failure (2, 5, 11, 13). Prenatal manifestations can include fetal hydrops, cardiomegaly, and myocardial dysfunction (7, 11, 14). Recent research reveals that hearing loss is a common extravascular manifestation in patients with *ENPP1*-deficient GACI, with a prevalence potentially exceeding 50% (1, 3, 10). Conductive hearing loss predominates (80%), likely due to ossicular chain (e.g., malleus, incus) discontinuity or fixation caused by abnormal mineralization (10). The incidence of recurrent otitis media is also significantly higher than in the general population, further exacerbating hearing damage (10). Sensorineural hearing loss is relatively less common and may be associated with calcification of the vessels supplying the inner ear (3, 10).

The pathogenesis of GACI is complex, with the core issue being an imbalance between extracellular calcification inhibitors and promoters. The PPi/Pi imbalance is the most widely accepted mechanism. Functional defects in *ENPP1* or *ABCC6* lead to decreased levels of extracellular PPi, a potent mineralization inhibitor (2, 3, 5). Concurrently, compensatory elevation of Fibroblast Growth Factor 23 (FGF23), while causing hypophosphatemia, may upregulate tissue-nonspecific alkaline phosphatase (TNAP). TNAP further hydrolyzes the already insufficient PPi into inorganic phosphate (Pi, a pro-mineralization factor), exacerbating the Pi/PPi ratio imbalance and driving the abnormal deposition of hydroxyapatite crystals in the vessel wall (1–3). Autopsy pathology in some infants shows early fragmentation of the arterial internal elastic lamina, with calcium salts deposited along the broken elastic fibers (4, 15). This could be a primary event or could be induced by the local inflammatory microenvironment (e.g., activation of matrix metalloproteinases by tumor necrosis factor-alpha) (4). Vascular stenosis is not caused by calcification alone. The AMP deficiency (and subsequently adenosine) production resulting from the *ENPP1* defect may directly stimulate vascular smooth muscle cell proliferation, causing significant intimal hyperplasia. This is a key factor leading to severe luminal stenosis or even occlusion (2, 4). Infiltration of T lymphocytes and macrophages can be observed around calcified lesions, suggesting inflammatory involvement in the disease process (4). Heterotopic ossification may even occur in advanced lesions (5).

The prognosis of GACI is extremely poor, with a very high early mortality rate. The vast majority of deaths occur within the first 6 months of life, common causes include myocardial infarction, intractable heart failure, malignant hypertension, and multi-organ failure (2, 5, 16). Retrospective studies indicate that approximately 55% of infants die during infancy (1–3). Survival rates improve significantly if the child survives this “critical period” of about 6 months (2, 3), with several reports of long-term survival into the 20s (17, 18). However, survivors face long-term challenges, including persistent hypertension, progressive vascular stenosis (even if calcification regresses), hypophosphatemic rickets (Autosomal Recessive Hypophosphatemic Rickets type 2, ARHR2), periarticular calcifications, progressive hearing loss, cervical spine fusion, and retinopathy (PXE-like changes), all of which severely impact quality of life (1–3).

At present there is no cure for GACI. Treatment is supportive and symptomatic, aiming to stabilize the condition, alleviate symptoms, and prolong survival. Bisphosphonates are the most commonly used drugs, particularly first-generation non-nitrogen-containing bisphosphonates like etidronate, due to their structural similarity to PPi and theoretical ability to inhibit ectopic mineralization. However, their efficacy is highly controversial. Early retrospective studies suggested a potential association with improved survival (5, 16), but recent analyses indicate they may not improve long-term prognosis and carry risks of skeletal toxicity (e.g., osteomalacia-like changes) with long-term use (2, 3, 19). Nitrogen-containing bisphosphonates (e.g., pamidronate, zoledronic acid), which potently inhibit bone resorption rather than mineralization, are theoretically less ideal choices (2, 3). Sodium thiosulfate, a calcium chelator, has shown potential to reduce calcification in individual case reports but is ineffective against vascular stenosis and may cause side effects like metabolic acidosis (5, 12, 19). Standard cardiovascular medications for managing hypertension and heart failure are crucial. Recombinant *ENPP1*-Fc Enzyme Replacement Therapy shows promise in animal models for simultaneously inhibiting calcification and intimal hyperplasia. This therapy is the most hopeful targeted therapy and has entered early-stage clinical trials (2, 3, 5). Oral PPi supplementation is another potential strategy (2, 3). Additionally, in non-skeletal tissues, TNAP may regulate extracellular PPi levels by hydrolyzing PPi, thereby preventing ectopic calcification in blood vessels and soft tissues. Therefore, TNAP inhibitors could be an important therapeutic strategy for increasing endogenous PPi and preventing ectopic soft tissue calcification in GACI patients (20). However, TNAP inhibitors are still in the early stages of clinical research. For end-stage heart failure, heart transplantation is the final treatment option, with successful cases reported (2, 5). For specific vascular stenosis, angioplasty or bypass surgery may be required (2).

Early and accurate diagnosis is crucial for improving management and genetic counseling. Prenatal diagnosis relies primarily on fetal ultrasound. Characteristic findings include: hyperechogenicity of large artery walls (especially the aorta and outflow tracts), fetal hydrops, polyhydramnios, pericardial/pleural effusion, cardiomegaly, and cardiac dysfunction (7, 8, 11, 14, 15). Those with a positive family history are key screening targets. Imaging is the cornerstone of Postnatal clinical diagnosis. Ultrasonography/Echocardiography: Non-invasive assessment of cardiac function, vessel wall echogenicity, and stenosis; the preferred screening and follow-up tool (6, 12). Computed Tomography (CT) and CT Angiography (CTA): The gold standard imaging methods for evaluating the extent and degree of systemic arterial calcification and luminal stenosis, essential for diagnosis and follow-up (5, 6, 19). Chest X-ray has low sensitivity and is prone to missed diagnosis (3, 6). The sequencing analysis of genes like *ENPP1* and *ABCC6* is the key method for definitive diagnosis, clarifying the genetic etiology and guiding family genetic counseling and prenatal diagnosis (5, 8, 9, 16). Arterial biopsy showing hydroxyapatite calcification in the internal elastic lamina and media with intimal hyperplasia is the pathological gold standard for diagnosis. However, due to its invasive nature, it is primarily performed during autopsy or under special circumstances (9, 15, 16).

GACI is a rare and severe genetic disease that requires lifelong multidisciplinary follow-up and management, posing great challenges to clinical diagnosis and treatment as well as family genetic counseling.

## Conclusion

GACI is a catastrophic disease caused by mutations in genes, such as *ENPP1* or *ABCC6*, characterized by systemic arterial calcification and intimal hyperplasia. The clinical manifestations of GACI prominently include refractory hypertension, heart failure, and hearing impairment. The primary pathological mechanism involves an imbalance in mineralization due to PPi deficiency. Despite recent advances in diagnosis (particularly prenatal ultrasound and genetic testing) and an improved understanding of its disease spectrum (including its overlap with PXE and ARHR2), treatment options remain very limited and controversial, leading to a poor prognosis and immense challenges for affected patients’ families and society. There is an urgent future need for prospective studies to clarify the disease course and to actively advance the clinical translation of targeted therapies like ENPP1 enzyme replacement to improve the survival quality and outcomes for patients with this rare disease.

## Data Availability

This study is a descriptive case report, and all clinical data presented are shown within the article and do not involve any database. The remaining medical records are stored in the Medical Records Department of Peking University International Hospital and can only be accessed by the patient themselves.
